# P01.38. Anti-cancer activity of extracts from Rauwolfia vomitoria and Pao Pereira

**DOI:** 10.1186/1472-6882-12-S1-P38

**Published:** 2012-06-12

**Authors:** J Yu, J Drisko, Q Chen

**Affiliations:** 1 University of Kansas Medical Center, Kansas City, USA

## Purpose

To evaluate extracts from two medical plants Pao Pereira (Pao) and Rauwolfia vomitoria (Rau) for their anti-tumor effects in various types of pancreatic cancers and ovarian cancers.

## Methods

Five pancreatic cancer and three ovarian cancer cell lines were tested that exhibited different resistance to the 1st line chemo-drug gemcitabine (Gem, for pancreatic cancer), and carboplatin (Cp, for ovarian cancer). Chou-Talalay's method was used to evaluate drug combination.

## Results

Both Rau and Pao extracts induced dose-dependent cytotoxicities in all tested cancer cell lines, despite their inherent resistance to chemo-drugs. IC_50_ values for Rau were 140-350µg/ml, and 120-350µg/ml for Pao, depending on the cells tested. Normal epithelial cell MRC-5 was much less affected compared to all the tested cancer cells. The differences of cell viabilities between cancer cells and normal cells were statistically significant (p<0.05), indicating possible low toxicity of these extracts. To test whether the treatments of Rau or Pao could enhance the cells’ sensitivities to chemo-drugs, we combined either Rau or Pao with gemcitabine to treat pancreatic cancer cells, and with carboplatin to treat ovarian cancer cells. The combination treatments took Chou-Talalay’s constant ratio design, with molar ratio set to IC_50extract_: IC_50Chemo_. The combined-treatments significantly enhanced cell death in cancer cells which were strongly resistant to gemcitabine or carboplatin (p<0.05). The results showed a left-shift in the dose-response curves of the combination treatments compared to the corresponding curves with either Gem or Cp alone in all tested cancer cells. Combination indices (CIs) were <1, indicating synergistic effects.

## Conclusion

These results pave the way for *in vivo* studies of the anti-cancer effects of Rauwolfia vomitoria and Pao Pereira extracts, especially in gemcitabine-resistant pancreatic cancers and carboplatin-resistant ovarian cancers. Studies on mechanisms of the anti-cancer actions are also undergoing concerning apoptosis and cell cycle arrests.

